# Associations of polygenic risk scores for type 2 diabetes with metabolic measures in Pacific Islanders from Guam and Saipan

**DOI:** 10.1038/s10038-026-01474-x

**Published:** 2026-04-09

**Authors:** Maria J. Ramirez-Luzuriaga, Saied Safabakhsh, Rasol Salehi, Sayuko Kobes, Tanisha F. Aflague, Jenny Duenas Sarmiento, Joanne E. Curran, Robert G. Nelson, Jeffrey M. Curtis, Carol D. Moffett, Wen-Chi Hsueh, Robert L. Hanson

**Affiliations:** 1 Phoenix Epidemiology and Clinical Research Branch National Institute of Diabetes and Digestive and Kidney Diseases, Phoenix, AZ USA; 2 Micronesian Institute for Disease Prevention and Research, Sinajana, Guam; 3 South Texas Diabetes and Obesity Institute, University of Texas Rio Grande Valley, Brownsville, TX USA; 4 Present Address: Pediatric Inherited Disease Research Center, Isfahan University of Medical Sciences, Isfahan, Iran; 5 Present Address: Research Division, Joslin Diabetes Center, Boston, MA USA; 6 Present Address: Division of Metabolism, Endocrinology and Nutrition, University of Washington, Seattle, WA USA

**Keywords:** Population genetics, Type 2 diabetes

## Abstract

Several polygenic risk scores (PRSs) for type 2 diabetes (T2D) have been derived from genome-wide association studies (GWASs), but Pacific populations have generally not been represented in these GWASs. The aim of this study was to examine the association of PRS for T2D with diabetes and clinical measures in Pacific Islander populations from Guam and Saipan. We analyzed seven constructions of PRSs in 1990 participants in a GWAS from a community-based cross-sectional study of diabetes. Associations of T2D PRS with diabetes, maximum body mass index (BMI), fasting glucose, and HbA1c were examined with adjustment for age, sex, and the first four genetic principal components (PCs) from the GWAS (to account for population stratification). Insulin resistance/secretion measures were also analyzed in 989 participants without diabetes. All seven PRSs were strongly associated with T2D (*p* < 5 × 10^−9^ for each); a 1-SD increase in the PRS was associated with 39–81% increase in the odds of T2D (i.e., standardized odds ratio [OR] ranging 1.39–1.81) adjusting for age, sex, and PCs. We also found that a 1-SD increase in the PRS was associated with 0.30–0.46 mmol/L higher fasting glucose, and with 2.68–4.63 mmol/mol higher HbA1c adjusting for age, sex, and PCs. We found no associations of PRSs with maximum BMI or insulin measures. All seven of the T2D polygenic risk scores evaluated associated significantly and strongly with diabetes, fasting glucose and HbA1c. These analyses suggest that T2D polygenic scores, as currently derived, have a certain degree of transferability to individuals from Pacific populations.

## Introduction

**Fig. 1:**
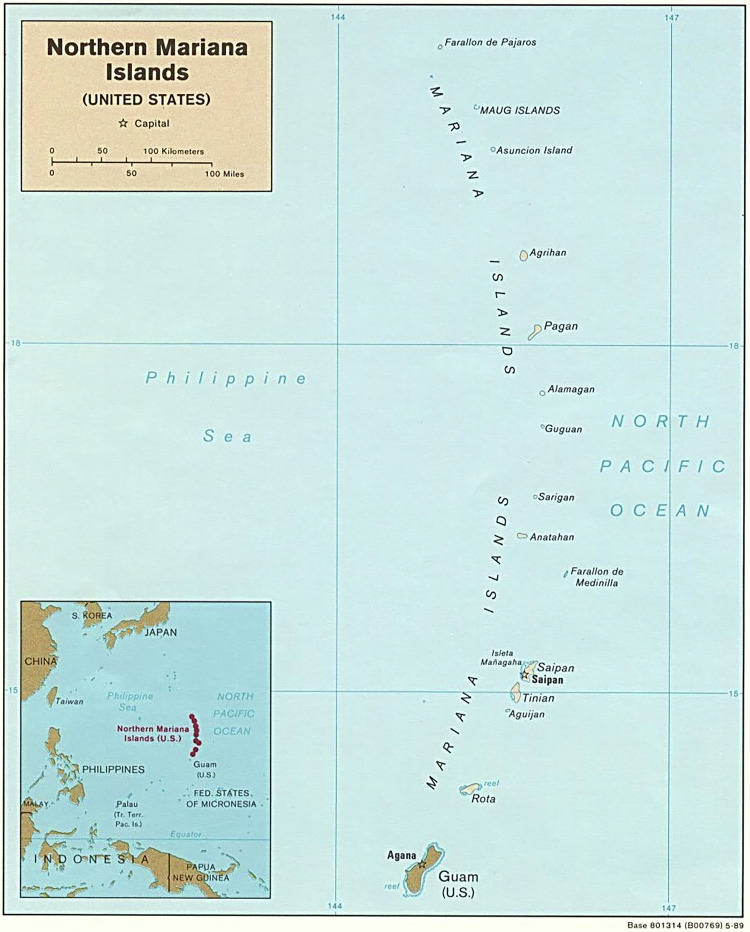
Map of the Mariana Islands showing the major islands of this archipelago in the North Pacific

Several polygenic risk scores (PRSs) for type 2 diabetes (T2D) have been derived from genome-wide association studies (GWASs), predominantly conducted in European-ancestry populations. There is limited information on how PRSs - based on variants from GWASs of T2D—transfer to Pacific Islander populations, which generally have not been represented in these GWASs, despite their high prevalence of T2D. Performance of PRSs often decreases when polygenic scores are applied to populations different from which they were derived, particularly when genetic differences between populations are large [1, 2]. In addition, genetic and environmental factors limit the transferability of PRSs across populations [3]. The present study evaluates established PRSs for T2D, constructed using publicly available GWAS summary statistics, in a Pacific Islander population with genotypic data who are at high risk of diabetes. We aimed to examine associations of T2D PRSs with diabetes, and with clinical measures in Pacific Islander populations from the Mariana Islands of Guam and Saipan (Fig. 1).

## Materials and methods

### Study design and participants

This study is derived from the Micronesian extension of the Family Investigation of Nephropathy and Diabetes (FIND) study, a community-based cross-sectional study of diabetes designed to identify genetic determinants of diabetes and kidney disease [4, 5]. The study was conducted by the National Institute of Diabetes and Digestive and Kidney Diseases in collaboration with the Micronesian Institute for Disease Prevention and Research. Study participants were recruited in 2008 and 2009 from dialysis centers, referrals and general advertising. Recruitment focused on individuals with diabetes and kidney disease, but the study was open to anyone who was at least 18 years old. The present sample consisted of 1990 study participants for whom genotypic data were available. The self-identified ethnic background for the largest number of participants was CHamoru (or Chamorro), the indigenous inhabitants of the Mariana islands, followed by Filipino and some other non-CHamoru Pacific populations. Plasma glucose was measured after ≥ 8 h fast in participants not known to have diabetes. Participants with known diabetes were not required to fast. Although no autoimmune testing was performed in this population, clinical characteristics suggest that most diabetes cases were type 2. Among participants with diabetes, 95% reported a disease history indicative of an absence of insulin dependence: they had either never used insulin, initiated insulin therapy more than one year after diagnosis, or experienced a period of at least one month without insulin use. Diabetes was defined as fasting plasma glucose ≥ 7.0 mmol/l, HbA1c ≥ 6.5% (48 mmol/mol) or a previous clinical diagnosis [6]. Fasting serum insulin concentrations were measured in participants without diabetes and insulin resistance and beta cell function were estimated according to the homeostatic model (HOMA-IR and HOMA-B) [7]. Maximum BMI was calculated from participant’s self-report of maximum body weight and height at this weight. Characteristics of study participants are shown in Table 1 and, according to diabetes status in Supplementary Table 1.

**Table 1:** Characteristics of participants (Mean ± SD or % unless otherwise specified) from Guam and Saipan with GWAS genotypes and clinical measures

	*n*	All participants
Age, years	1990	43.4 ± 17.1
Body Mass Index ^a^, kg/m^2^	1933	33.2 ± 8.65
Fasting plasma glucose, mmol/L	1414	6.36 ± 3.18
HbA1c, mmol/mol	1641	49.6 ± 22.5
HbA1c, %	1641	6.69 ± 2.05
HOMA-IR, μIU/mL*mmol/L ^b^	989	2.59 (1.58, 4.66)
HOMA-β (%)^b^	988	135.7 (82.4, 232.1)
Diabetes ^c^, %	1851 (761)	41.1
End Stage Renal Disease ^d^, %	1990 (327)	16.4
Primary Ethnicity ^e^		
CHamoru	905	45.5
Chuukese	173	8.69
Filipino	248	12.5
Other	664	33.3

^a^Body mass index was determined by self-reported maximum weight and corresponding height

^b^Values are reported as median (p25, p75)

^c^Diabetes was defined as fasting plasma glucose ≥ 7.0 mmol/l, HbA_1c_ ≥ 6.5% (48 mmol/mol) or a previous diagnosis of the condition

^d^End-stage renal disease was defined as need for dialysis

^e^Ethnicity was determined by self-report

The study was approved by the Institutional Review Boards of the National Institutes of Health and of the Guam Memorial Hospital Authority, and by the Commonwealth Healthcare Corporation of the Commonwealth of the Northern Mariana Islands. All participants provided written informed consent.

### Genotypes

Genotypic data were generated for the GWAS on the Illumina OmniExpress Core Exome array (Illumina, San Diego, CA). Genotypes were thus obtained for 260,761 single nucleotide polymorphisms (SNPs) that passed quality control metrics (call rate > 95%, minor allele frequency > 0.01, discrepancy rate < 2.5% in 100 blind duplicate samples, *p* > 0.0001 for Hardy-Weinberg proportions). Whole genome sequence data (BGI, Shenzhen, Guangdong, China) were also obtained for a subset of 40 full-heritage (by self-report) CHamoru. These sequence data were combined with those from Phase 3 of the 1000 Genomes Project (*n* = 2504) [8] to create a reference panel by use of a cross-imputation procedure [9]. Genotypes from this reference panel were imputed into the GWAS participants; phasing and imputation were conducted with BEAGLE (http://faculty.washington.edu/browning/beagle/beagle.html; version 5.2) [10, 11]. Imputed genotypes were thus generated for 8,240,479 additional SNPs with minor allele frequency > 0.01 and imputation r^2^ > 0.5. A subset of the GWAS-derived genotypes from the current Pacific Islander samples (19,969 SNPs) was also used in factor analysis to derive genetic principal components for use as covariates to control for population stratification.

### Construction of type 2 diabetes PRSs

PRSs for T2D were derived from variants identified in GWASs for which imputed genotypic data were available for the Pacific Islander study population. The following seven PRSs were constructed using summary statistics from publicly available GWASs, each named after the meta-analysis from which it was derived: (1) Khera 2018 (PGS000014, 6.9 M variants accounting for LD by LDPRED, a method which accounts for linkage disequilibrium among markers, in European ancestry GWASs) [12]; (2) Ge 2022 (PGS002308, composed of 1.2 M variants derived from PRS-CSX—a Bayesian method that estimates a threshold for how many variants to include, and which leverages differing allele frequencies and varying LD across ancestral populations to assign a weight for each variant included) [13]; (3) Mars 2022 (PGS002771, 1.1 M variants derived in European ancestry via PRSCS) [14]; (4) Prive 2022 (PGS001818, 30,745 variants LD by LDPRED derived from European-ancestry populations from the UK Biobank) [15]; (5) Suzuki 2024 (1289 variants derived by pruning and thresholding of genome-wide significant variants from a multi-ancestry meta-analysis) [16]; (6) The Diabetes Genetics Replications And Meta-analysis consortium (DIAGRAM) 2018 (PGS003729, 393 variants derived by pruning and thresholding of genome-wide significant variants from European populations) [17]; (7) Diabetes Meta-Analysis of Trans-Ethnic association studies consortium (DIAMANTE) 2022 (PGS003730, 338 variants derived from pruning and thresholding in multi-ancestry meta-analysis) [18]. Characteristics of these PRSs are shown in Table 2. PRSs were calculated by multiplying the number (i.e., the “dosage” from the imputation) of risk alleles by the absolute value of the weight (logarithm of the odds ratio) for each variant in the PRS, adding across all variants and dividing the total by the sum of the weights (so that scores are expressed per average number of risk alleles accounting for the weights). A small number of variants present in Khera 2018 and DIAGRAM 2018 PRSs were multiallelic in the reference panel; in these cases, any allele other than the designated risk allele in the PRS was considered “non-risk”. Resulting PRSs were standardized and Odds Ratios (ORs) and β coefficients for polygenic scores were expressed in terms of SD of that PRS.

**Table 2:** Polygenic risk scores for type 2 diabetes analyzed for association with diabetes and clinical measures

Reference	PGS catalog #	Ancestry	Method ^a^	# Variants in GWAS	# Variants imputed in target population	Effective sample size^b^
Khera, [12]	PGS000014	European	LDPRED	6.9 M	5.5 M	44 K
Ge, [13]	PGS002308	Multiple	PRS-CSX	1.2 M	1.1 M	239 K
Mars, [14]	PGS002771	European	PRSCS	1.1 M	1 M	136 K
Prive, [15]	PGS001818	European	LDPRED	30,745	30,544	35 K
Suzuki, [16]	–	Multiple	P&T	1,289	1,122	712 K
DIAGRAM, [17]	PGS003729	European	P&T	393	321	136 K
DIAMANTE, [18]	PGS003730	Multiple	P&T	338	315	313 K

^a^P&T is pruning and thresholding

^b^Effective sample size is 2/[(1/n_cases)+(1/n_controls)] [33]

### Statistical analyses

Association of PRSs with diabetes were analyzed by logistic regression. We estimated several effect measures: (a) strength of association (standardized odds ratio and pseudo-R^2^), a general measure of variance explained for logistic regression models, calculated by Nagelkerke’s method [19]; (b) area under the receiver operating characteristic curve (AUC) to measure the performance of models to distinguish between individuals with and without diabetes, based on adding the PRS to the model containing covariates only [20]; and (c) Cochran’s Q test to calculate the significance of differences between odds ratios [21]. For comparing PRSs, standard errors for differences in AUCs for pairs of PRSs were calculated by a bootstrap procedure. We used linear regression models to examine associations of PRS with maximum BMI, fasting glucose and HbA1c, in all participants irrespective of diabetes status. Associations of PRS with measures of insulin resistance and beta-cell function (HOMA-IR, HOMA-B) were conducted in participants without diabetes. All models were adjusted for age, sex and the first four genetic Principal Components (PCs) from the GWAS, to account for potential population stratification. In separate models we also accounted for maximum BMI and conducted analyses excluding participants with kidney failure, defined as the need for dialysis or transplant, to assess whether their inclusion influenced the results. We also analyzed models that were stratified by ethnicity. For these analyses we divided individuals into four groups: those whose self-reported ancestry was full-heritage CHamoru, full heritage Filipino, full heritage Chuukese and others (66% of this group reported more than one ancestry, and 55% reported partial CHamoru ancestry). Analyses were conducted using SAS 9.4. Although some individuals in the study were related, we did not account for this in the primary analyses, as such information may not be routinely available in clinical situations. In sensitivity analyses, we also ran models fitted with a variance components covariance structure to account for genetic relatedness among individuals, which was estimated with PREST [22]. These analyses were conducted using SOLAR, with diabetes status treated as a (0,1) quantitative trait [23]. Regression coefficients were converted to odds ratios by the method of Haggstrom [24].

In the primary analyses, we adjusted for the PCs derived from the present GWAS. However, in clinical situations, such population-specific PCs may not be readily available. We, thus, also conducted analyses in which we performed an “ad hoc” adjustment to correct for PCs derived from the 1000 Genomes project, projected into the current samples [described in Ge, ref. [12]]. PCs for the subset of 19969 SNPs used for the analyses in the current sample were generated in the 2504 individuals from the 1000 Genomes dataset. The first 4 PCs derived in this analysis largely separated the five major continental superpopulations in the 1000 Genomes data. These 4 PCs were regressed against the PRS calculated in the 1000 Genomes samples. These 4 PCs were also calculated in the current Pacific Islander samples by applying the factor pattern coefficients derived in the 1000 Genomes data to the relevant SNPs. An adjusted PRS was then calculated by applying the regression coefficients calculated in the 1000 Genomes data to these PCs projected from the 1000 Genomes into the current data. By this procedure a PRS, adjusted for the 1000 Genomes global ancestry estimates, can be calculated for any sample without the need to generate local population-specific PCs. The first PC distinguished African (AFR) from non-African populations and PC2 differentiated East Asian (EAS) and European (EUR) populations. Individuals from the present study, whom we characterize as “Mariana Pacific Islanders” (MPI, which includes Pacific Islanders and Filipinos), project between EAS and EUR but cluster closer to EAS (Supplementary Fig. 1). To assess genetic distances, we also estimated F_ST_ between populations across the markers used to calculate the PCs [25]. These estimates indicate that MPI are most closely related to EAS populations (F_ST_ = 0.018), with greater differentiation from EUR (F_ST_ = 0.085) populations, while F_ST_ between EAS and EUR was 0.105 (Supplementary Table 2).

### Comparison with ARIC study

For comparison of association results with non-Pacific cohorts, we downloaded data for the Atherosclerosis Risk in Communities (ARIC) Study from the database for Genotype and Phenotype (dbGAP) resource (dbGAP accession phs.000280.v7.p1, accessed January 4, 2023) [26]. Diabetes was defined as a documented history of diabetes or fasting plasma glucose ≥ 7.0 mmol/l. Analyses were conducted in 9285 European American participants (1952 with diabetes) with genotypic data using the same methods as in the Pacific Islander studies. European Americans from ARIC were chosen as a comparison population because their data were not used in most of the GWASs used to derive the PRSs. Since standard deviations of the PRSs may differ across populations, we express the odds ratio for a constant difference in number of risk alleles (e.g., for a difference of 5000 risk alleles), rather than per SD, when comparing odds ratios across populations.

### Differences in PRS across populations

Since allele frequencies and linkage disequilibrium patterns can vary across ancestral groups, PRSs developed in one population may not perform as well or have the same variance characteristics when applied to another population. Such differences across populations can present challenges in subsequent analyses or comparisons. We examined differences in the distribution of PRSs between our sample population (e.g., MPI) and the five superpopulations defined by the 1000 Genomes Project: Africans (AFR), Admixed Americans (AMR), East Asians (EAS), Europeans (EUR), and South Asians (SAS). To quantify the differences in PRS across populations we calculated the phenotypic differentiation coefficient (V_ST_), which represents the proportion of the total variance in the PRS explained by subpopulation membership [27, 28]; V_ST_ is analogous to F_ST_ (the co-ancestry coefficient), which is commonly used to quantify genetic differentiation among populations. In addition to the raw PRS values, we also calculated V_ST_ with adjustment for the first four 1000 Genomes PCs to determine the extent to which this adjustment reduces differences among groups. We further calculated V_ST_ among the subgroups in the current study (i.e., CHamoru, Filipino, Chuukese, and others). Confidence intervals for V_ST_ were calculated by a bootstrap method.

## Results

### Associations of T2D polygenic scores with diabetes

**Fig. 2:**
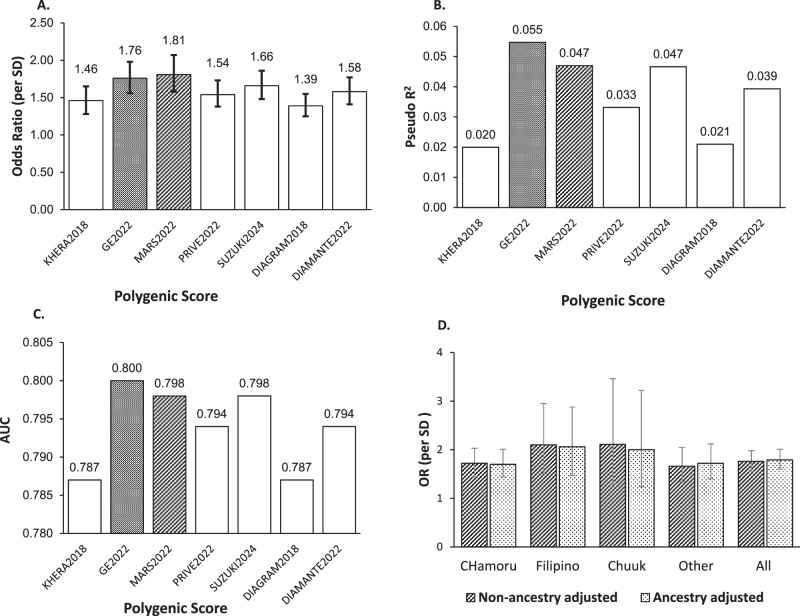
**A** Odds ratios (per SD) of associations of polygenic scores with type 2 diabetes. Covariates include age, sex and first four principal components. **B** Pseudo-R^2^ of polygenic scores by Nagelkerke’s method. **C** Area under the receiver operating characteristic curve (AUC) of polygenic scores. **D** Association of Polygenic score GE2022 (Ge et al. Genome Med, 2022) with type 2 Diabetes by ethnicity. Values are odds ratios (per 5000 weighted risk alleles) adjusted for age, sex and study-specific PC. Ancestry-adjusted OR were, alternatively, corrected for projected PCs derived from the1000 Genomes Project

All seven of the polygenic risk scores for T2D were strongly and significantly associated with diabetes in this Pacific Islander population (Fig. 2A). Odds Ratios for the polygenic risk scores in models adjusted for age, sex, and the first four PCs ranged from 1.39 to 1.81 per SD (*p* = 3.7 × 10^−9^ to 6.9 × 10^−23^). Results remained statistically significant in models additionally adjusting for maximum BMI and in models excluding participants with kidney failure (Supplementary Table 3). Among the seven different PRSs, the score composed of ~1.1 M variants, derived from a European GWAS by PRS-CSx (PGS002771 Mars, *Am J Hum Genet*, 2022) provided the strongest associations with T2D, as measured by the standardized OR (1.81, 95% CI, 1.58–2.07) (Fig. 2A). However, the score composed of 1.2 M variants, derived from a multi-ancestry GWAS by PRS-CSx (PGS002308 Ge, *Genome Medicine*, 2022) provided the strongest association with T2D and the best predictive accuracy, as assessed by R^2^ and AUC measures (Fig. 2B, C). The PRS from the multi-ancestry GWAS by Suzuki (*Nature*, 2024, 1289 variants) had standardized OR, R^2^ and AUC values that were only slightly below that for the Ge and Mars PRSs. We found similar results in analyses additionally accounting for the genetic relatedness among individuals (Supplementary Table 4). When comparing the area under the receiver operating characteristic curve between polygenic scores for T2D derived by Mars et al. (PGS002771, *Am J Hum Genet*, 2022) and Ge et al. (PGS002308, *Genome Medicine*, 2022), differences in AUC were not statistically significant (*p* value = 0.439) (Table 3). For PRSs derived using data from Ge 2022, Mars 2022 and Suzuki 2024, adjustment for projected PCs derived from the 1000 Genomes produced similar results to those adjusted for study-specific PCs (Supplementary Table 5).

**Table 3:** Area under the operating curve (AUC) comparisons between models examining associations of polygenic risk scores with type 2 diabetes

	AUC	Khera [12]	Ge [13]	Mars [14]	Prive [15]	Suzuki [16]	DIAGRAM [17]	DIAMANTE [18]
**Khera** [12]	0.787	–	0.001	0.001	0.084	0.012	1.00	0.084
**Ge** [13]	0.800	0.013	–	0.439	0.144	0.546	<0.001	0.079
**Mars** [14]	0.798	0.011	0.002	–	0.295	1.00	0.002	0.298
**Prive** [15]	0.794	0.007	0.006	0.004	–	0.382	0.073	1.00
**Suzuki** [16]	0.798	0.011	0.002	0.000	0.004	–	0.002	0.214
**DIAGRAM** [17]	0.787	0.000	0.013	0.011	0.007	0.011	–	0.023
**DIAMANTE** [18]	0.794	0.007	0.006	0.004	0.000	0.004	0.007	–

AUC *p* values above diagonal, absolute value of AUC difference below diagonal. AUC Models adjusted for age, sex and the first four genetic PCs. The AUC for the model with covariates only is 0.778

For all constructions of PRSs, OR estimates for association between T2D polygenic scores and diabetes were higher in Chuukese and Filipinos than in the other study populations. However, confidence intervals overlapped between groups, and none of the differences were statistically significant (Supplementary Table 6). Correction for projected PCs derived from 1000 G also resulted in similar differences in odds ratios across ethnic groups (Fig. 2D, Supplementary Table 5).

### Comparisons with European Americans from ARIC

Differences between associations of PRSs with T2D obtained in the Pacific Islander+Filipino population, and those obtained in European Americans from the ARIC study are shown in Supplementary Tables 7 and 8. In general, differences in ORs and AUCs were similar between the Pacific Islanders and European Americans and ORs were not statistically significantly different; pseudo R^2^ values were generally moderately higher in European Americans. For instance, for the Ge 2022 PRS, the OR for a difference of 5000 risk alleles was 2.19 (95% CI, 1.85–2.58) in Pacific Islanders. For comparison, the OR for a 5000-risk allele difference in European Americans from the ARIC study was 2.34 (95% CI, 2.17–2.52). The *p* value for heterogeneity was 0.48.

### Differences in PRS distribution across populations

**Fig. 3:**
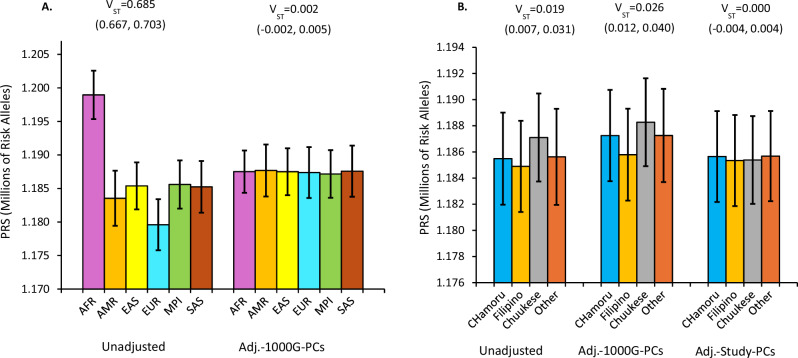
**A** Distribution of Ge 2022 PRS across the current sample (MPI) and 1000 Genomes populations. Data represent the mean value (±1 SD) of the PRS for each population. V_ST_ is the phenotypic differentation coefficient (with 95% confidence intervals). **B** Distribution of Ge 2022 PRS-Guam. Data represent the mean value (±1 SD) of the PRS for each population. V_ST_ is the phenotypic differentation coefficient (with 95% confidence intervals). AFR African AMR Admixed American, EAS East Asian EUR European, MPI Mariana Pacific Islander, and SAS South Asians

For all PRSs, there were significant differences in distribution among the populations constituted by our study population and the five 1000 G superpopulations; the biggest differences in the distribution of risk alleles between Pacific Islanders + Filipinos and the five superpopulations were generally observed with Africans or Europeans, while the smallest differences were with East Asians or South Asians. For the Ge 2022 PRS, for example, 68.5% of the variance in PRS in the combined sample was explained by population differences and the difference in mean PRS between Pacific Islanders and Africans was 3.71 SD; adjustment for the 1000 G PCs reduced these differences substantially (Fig. 3A). Similar results were seen with the other PRSs (Supplementary Fig. 2–7). Differences among Pacific Islander population subgroups (i.e., CHamoru, Filipino, Chuukese, and others) were much more modest, but in some cases were still statistically significant. For the Ge 2022 PRS, 1.9% of the variance in Pacific Islanders was explained by subpopulation, with the largest difference between populations being 0.64 SD, between Filipinos and Chuukese (Fig. 3B). These differences were not reduced by adjustment for the 1000 G PCs, but they were reduced with adjustment for study-specific PCs. Results for the other PRSs were generally similar (Supplementary Figs. 8–13).

### Associations of T2D polygenic scores with clinical measures

All seven constructions of PRS for T2D were significantly associated with fasting plasma glucose and with HbA1c among all participants. We found that 1-SD increase in the PRS was associated with 0.30–0.46 mmol/L higher fasting glucose, and with 2.68–4.63 mmol/mol increase in HbA1c adjusting for age, sex, and PCs (Table 4). Results remained significant when participants with kidney failure were excluded (Supplementary Tables 9 and 10). In analyses excluding participants with diabetes, associations between PRS and fasting plasma glucose were no longer statistically significant. However, associations between PRS and HbA1c remained significant for all PRSs except those derived from Khera 2018 and DIAGRAM 2018 (Supplementary Table 11).

**Table 4:** Associations of T2D polygenic scores with clinical measures (per 1 SD increase in PRS)^a^

	Maximum BMI, kg/m^2^ *n* = 1933		Fasting plasma glucose, mmol/L *n* = 1414		HbA1c, mmol/mol *n* = 1641		HOMA-IR, μIU/mL*mmol/L *n* = 989		HOMA-B, μIU/mL *n* = 988	
	β (95% CI)	*p* value	β (95% CI)	*p* value	β (95% CI)	*p* value	β (95% CI)	*p* value	β (95% CI)	*p* value
Khera [12]	0.19 (−0.23, 0.62)	0.379	0.30 (0.10, 0.49)	0.002	2.87 (1.65, 4.09)	<0.001	1.01 (0.95, 1.07)	0.663	1.00 (0.94, 1.06)	0.898
Ge [13]	0.21 (−0.16, 0.59)	0.269	0.46 (0.28, 0.63)	<0.001	4.32 (3.26, 5.38)	<0.001	1.01 (0.95, 1.07)	0.611	0.98 (0.93, 1.03)	0.478
Mars [14]	0.26 (−0.16, 0.70)	0.223	0.46 (0.27, 0.66)	<0.001	4.63 (3.42, 5.84)	<0.001	1.03 (0.97, 1.10)	0.318	1.01 (0.95, 1.07)	0.831
Prive [15]	0.24 (−0.14, 0.62)	0.218	0.35 (0.18, 0.52)	<0.001	3.23 (2.16, 4.30)	<0.001	1.03 (0.98, 1.09)	0.258	1.02 (0.97, 1.07)	0.543
Suzuki [16]	0.09 (−0.27, 0.47)	0.608	0.44 (0.27, 0.60)	<0.001	4.20 (3.15, 5.25)	<0.001	1.02 (0.96, 1.07)	0.568	0.98 (0.93, 1.03)	0.467
DIAGRAM [17]	−0.06 (−0.43, 0.31)	0.744	0.30 (0.14, 0.47)	<0.001	2.68 (1.62, 3.74)	<0.001	0.98 (0.93, 1.03)	0.428	0.97 (0.92, 1.02)	0.244
DIAMANTE [18]	−0.02 (−0.39, 0.34)	0.888	0.42 (0.25, 0.58)	<0.001	3.95 (2.92, 4.99)	<0.001	1.00 (0.95, 1.06)	0.918	0.97 (0.92, 1.02)	0.179

^a^ Estimates adjusted for age, sex, and PCs

We found no associations of PRSs for T2D with maximum BMI (Table 4 and Supplementary Table 12), or with measures of insulin resistance and beta cell function in participants without diabetes (Table 4 and Supplementary Tables 13 and 14). In addition, we found no major differences in these associations when results were corrected for projected PCs derived from 1000 G instead of the study-specific PCs (Supplementary Table 15).

## Discussion

In this study, we assessed seven previously published T2D PRSs, using genotypic and phenotypic data of a Pacific Islander population from Guam and Saipan. Our sample consisted of 1990 genotyped individuals who were predominantly of CHamoru ancestry (the indigenous people of the Mariana Islands), but it also included Filipinos and other Pacific Islanders residing in Guam or Saipan. We first assessed the transferability of PGSs to the Pacific Islander population. We constructed the seven PRSs using GWAS data from the Pacific Islanders, and we assessed whether these scores were associated with diabetes, glycemic traits, and clinical measures. In models adjusting for age, sex and the first four genetic PCs, we found that all seven PRSs evaluated associated significantly and strongly with diabetes, fasting glucose and HbA1c, but not with maximum BMI or measures of insulin resistance and beta-cell function. In models excluding participants with diabetes, associations of PRSs with fasting glucose were no longer significant. However, associations of PRSs with HbA1c remained significant for most PRSs, suggesting that PRSs associate with glycemia in those without diabetes.

We also evaluated the performance of the PRSs by pseudo R^2^ and AUC. We found that all PRSs performed reasonably well, except those derived from European GWAS by Khera 2018 and DIAGRAM 2018, which consistently underperformed compared with the others. The relatively small sample sizes used in the derivation of these two scores may explain their underperformance. While the PRS derived by Khera 2018 was composed of 6.9 M variants, the effective sample size used in its derivation was 44 K, which is small relative to most of the other PRSs. Similarly, the number of variants used in the derivation of PRS by DIAGRAM 2018 was relatively small at 393 and it was derived solely from individuals of European ancestry (see Table 2). Notably, our analysis highlighted PGS002308 derived from multi-ancestry GWAS by PRS-CSx (Ge, *Genome Medicine*, 2022) and PGS002771 derived from European GWAS by PRSice (Mars, *Am J Hum Genet*, 2022) as particularly strongly associated with T2D. While PGS002771 provided the strongest associations with T2D as measured by standardized OR, PGS002308 provided the best predictive accuracy, as assessed by R^2^ and AUC. PGS002308 has previously demonstrated a significant association with T2D status across different ancestral groups (European, African, and Hispanic/Latino) [13]. Previous studies found that PRSs derived from multi-ancestry data often outperform those of single ancestries, particularly in diverse populations [1, 29]. PGS002308 was derived using multi-ancestry populations of European [17], African American [30], and Japanese descent [31], and was the PRS with the strongest association with T2D in our Pacific Islander population, highlighting its potential transferability. Nonetheless, these analyses suggest that PGS002771 provides comparable utility, as does the recent PGS derived by Suzuki et al. composed of 1289 independent genome-wide significant signals from a multi-ancestry GWAS [16]. Notably, the effect sizes (ORs) observed for these PRSs were comparable to, and not significantly different from those observed for individuals of European ancestry from the ARIC study. While prior reports suggest reduced PRS performance in East Asian populations [32], our population structure analyses indicate that Mariana Pacific Islanders (MPI) are genetically closer to East Asians than Europeans, based on PCA and F_ST_ estimates (see Supplementary Fig. 1 and Supplementary Table 2). The similar PRS performance between MPI and European Americans observed here is therefore likely influenced by factors such as population-specific linkage disequilibrium and allele frequency patterns, rather than genetic proximity to Europeans. However, with moderate sample sizes and high variability within populations, the power of the present analysis to detect modest differences in effect sizes may be limited.

While the strong associations, and similar effect sizes with T2D are indicative of potential transferability of PRSs to Pacific Islander populations, clinical translation may also need to consider differences in PRS distribution among ancestry groups. Such differences may reflect bias introduced by the selection of variants for GWAS arrays and by population differences in linkage disequilibrium with causal variants, in addition to differences in genetic disease risk among ancestry groups [33, 34]. When comparing the distribution of PRSs in our study population with the five superpopulations defined by the 1000 Genomes Project, we found substantial differences, particularly with African and European groups for all PRSs. For instance, for the PRS derived using data from Ge 2022, the mean difference in risk alleles of our study population with Africans and Europeans was 3.71 and 1.65 SD, respectively. These differences reduced substantially with adjustment for principal components projected from the 1000 Genomes Project; for the Ge 2022 PRS the differences were 0.10 and 0.06 SD for Africans and Europeans, respectively. This suggests that this “ad hoc” method can adjust for differences in PRSs among continental populations, and, thus, may facilitate interpretation of PRSs across populations. However, we also observed modest, but potentially meaningful, differences among Pacific Islander subpopulations for several PRSs. These differences were not reduced by adjusting for PCs projected from the 1000 Genomes but were reduced by adjusting for the study specific PCs. Since the 1000 Genomes data do not contain any samples from Pacific populations, adjustment for the 1000 Genomes PCs may not capture information about specific subpopulations in the current study. These findings indicate limited utility of adjusting for PCs projected from the 1000 Genomes in situations where there are individuals from different subpopulations, and it suggests that information on local genetic substructure, e.g., from the study-specific PCs, may be required for optimal interpretation of PRSs in these situations.

There are limitations to this study. The ancestry of the participants was largely CHamoru, with limited numbers from other Pacific populations, and may not be representative of Pacific populations as a whole. Study participants were recruited from dialysis centers, and participants with diabetes were also oversampled. Thus, the possibility of sample bias cannot be ruled out. However, analyses excluding participants with kidney failure showed similar results to those that included them. Given the sampling strategy, the prevalence of diabetes in our study population is probably not reflective to that in Guam and Saipan generally. Further population-based studies, preferably based on longitudinal data, are needed to accurately predict diabetes risk with PRSs, which, optimally, would include Pacific populations in their derivation. Additional work across multiple populations, in laboratory methods, and in assessing clinical benefits will also be needed before PRSs are incorporated into clinical care.

In conclusion, this study found that current T2D PRSs have transferability potential to Pacific Islanders from Guam and Saipan, given their strong associations with diabetes and similar effect sizes to those seen in European Americans from the ARIC study. This suggests that current PRSs can be used to rank individuals for diabetes risk within specific subpopulations, *e.g*., for clinical studies, but further work is needed to assess how accurately they can assess diabetes risk in Pacific populations.

## Supplementary information

Supplemental Material

## Data Availability

Data for consenting individuals will be made available through the Database of Genotype and Phenotype (https://www.ncbi.nlm. nih.gov/gap) pending Institutional Review Board approval.
