# Supplementary material for: Associations of polygenic risk scores for type 2 diabetes with metabolic measures in Pacific Islanders from Guam and Saipan
Source: J Hum Genet. 2026 Apr 9;71(8):493–500. doi: 10.1038/s10038-026-01474-x (PMC13400312; doi:10.1038/s10038-026-01474-x)
Supplement: Supplementary file 1 — Supplemental Material [file 10038_2026_1474_MOESM1_ESM.docx]

# **Supplemental Table 1.** Characteristics of participants (Mean ± SD or % unless otherwise specified) from Guam and Saipan with GWAS genotypes and clinical measures by diabetes status (n=1851) ^a^

|  | **Without diabetes**  **n=1090** | | **With Diabetes**  **n=761** | |
| --- | --- | --- | --- | --- |
|  | **n** |  | **n** |  |
| Age, years | 1090 | 36.9 ± 15.6 | 761 | 52.0 ± 14.6 |
| Body Mass Index, kg/m^2^ | 1058 | 31.5 ± 7.76 | 740 | 35.9 ± 9.26 |
| Fasting plasma glucose, mmol/L | 1090 | 5.27 ± 0.57 | 309 | 10.3 ± 5.08 |
| HbA1c, mmol/mol | 1090 | 38.6 ± 3.83 | 526 | 73.0 ± 27.3 |
| HbA1c, % | 1090 | 5.68 ± 0.35 | 526 | 8.83 ± 2.49 |
| HOMA-IR, μIU/mL*mmol/L ^b^ | 989 | 2.59 (1.58, 4.66) | 0 | - |
| HOMA-β (%) ^b^ | 988 | 135.7 (82.4, 232.1) | 0 | - |
| End Stage Renal Disease, % | 5 | 0.46 | 232 | 30.5 |
| Primary Ethnicity |  |  |  |  |
| CHamoru | 458 | 42.0 | 393 | 51.6 |
| Chuukese | 125 | 11.5 | 43 | 5.65 |
| Filipino | 119 | 10.9 | 95 | 12.5 |
| Other | 388 | 35.6 | 230 | 30.2 |

^a^ Out of 1990 study participants, 139 had genotypic data but missing information on criteria required to assess diabetes status using the ADA 2010 criteria.

^b^ Values are reported as median (p25, p75).

# **Supplemental Table 2.** Pairwise FST estimates based on the Mariana Pacific Islander samples (19969 SNPs) used for the 1000 Genomes Project principal component analysis.

|  | **MPI** | **EAS** | **SAS** | **AMR** | **EUR** | **AFR** |
| --- | --- | --- | --- | --- | --- | --- |
| **MPI** | - | 0.018 | 0.054 | 0.056 | 0.085 | 0.153 |
| **EAS** |  | - | 0.067 | 0.068 | 0.105 | 0.166 |
| **SAS** |  |  | - | 0.032 | 0.036 | 0.124 |
| **AMR** |  |  |  | - | 0.026 | 0.117 |
| **EUR** |  |  |  |  | - | 0.137 |
| **AFR** |  |  |  |  |  | - |

# **Supplemental Table 3.** Associations of polygenic risk scores with type 2 diabetes (OR per SD)

|  | **Excluding participants with kidney failure** | | | | **Including participants with kidney failure** | | | |
| --- | --- | --- | --- | --- | --- | --- | --- | --- |
|  | **n** | **OR** | **95% CI** | **p-value** | **n** | **OR** | **95% CI** | **p-value** |
| **Khera 2018 (AUC=0.787)** |  |  |  |  |  |  |  |  |
| Adjusted for age, sex, and PCs | 1614 | 1.42 | 1.24, 1.63 | <0.0001 | 1851 | 1.46 | 1.28, 1.65 | <0.0001 |
| Adjusted for age, sex, PCs, and max BMI | 1569 | 1.47 | 1.27, 1.70 | <0.0001 | 1798 | 1.50 | 1.31, 1.72 | <0.0001 |
| By ethnicity ^1^ |  |  |  |  |  |  |  |  |
| CHamoru | 716 | 1.34 | 1.11, 1.63 | 0.0022 | 851 | 1.34 | 1.12, 1.60 | 0.0010 |
| Filipino | 192 | 1.60 | 1.06, 2.40 | 0.0239 | 214 | 1.71 | 1.16, 2.53 | 0.0064 |
| Chuuk | 159 | 1.41 | 0.70, 2.84 | 0.3293 | 168 | 1.20 | 0.66, 2.18 | 0.5355 |
| Other | 547 | 1.60 | 1.25, 2.06 | 0.0002 | 618 | 1.63 | 1.29, 2.06 | <0.0001 |
| **Ge 2022 (AUC=0.800)** |  |  |  |  |  |  |  |  |
| Adjusted for age, sex, and PCs | 1614 | 1.64 | 1.44, 1.86 | <0.0001 | 1851 | 1.76 | 1.56, 1.98 | <0.0001 |
| Adjusted for age, sex, PCs, and max BMI | 1569 | 1.69 | 1.48, 1.94 | <0.0001 | 1798 | 1.83 | 1.62, 2.08 | <0.0001 |
| By ethnicity ^1^ |  |  |  |  |  |  |  |  |
| CHamoru | 716 | 1.63 | 1.35, 1.96 | <0.0001 | 851 | 1.72 | 1.45, 2.03 | <0.0001 |
| Filipino | 192 | 1.89 | 1.33, 2.69 | 0.0004 | 214 | 2.10 | 1.50, 2.95 | <0.0001 |
| Chuuk | 159 | 2.05 | 1.20, 3.51 | 0.0082 | 168 | 2.11 | 1.29, 3.46 | 0.0030 |
| Other | 547 | 1.54 | 1.22, 1.94 | 0.0002 | 618 | 1.66 | 1.34, 2.05 | <0.0001 |
| **Mars 2022 (AUC=0.798)** |  |  |  |  |  |  |  |  |
| Adjusted for age, sex, and PCs | 1614 | 1.70 | 1.47, 1.96 | <0.0001 | 1851 | 1.81 | 1.58, 2.07 | <0.0001 |
| Adjusted for age, sex, PCs, and max BMI | 1569 | 1.76 | 1.51, 2.04 | <0.0001 | 1798 | 1.88 | 1.63, 2.16 | <0.0001 |
| By ethnicity ^1^ |  |  |  |  |  |  |  |  |
| CHamoru | 716 | 1.73 | 1.40, 2.14 | <0.0001 | 851 | 1.78 | 1.47, 2.15 | <0.0001 |
| Filipino | 192 | 1.96 | 1.30, 2.96 | 0.0012 | 214 | 2.22 | 1.50, 3.29 | <0.0001 |
| Chuuk | 159 | 2.19 | 1.15, 4.18 | 0.0151 | 168 | 2.21 | 1.20, 4.07 | 0.0090 |
| Other | 547 | 1.55 | 1.20, 1.98 | 0.0006 | 618 | 1.67 | 1.32, 2.11 | <0.0001 |
| **Prive 2022 (AUC=0.794)** |  |  |  |  |  |  |  |  |
| Adjusted for age, sex, and PCs | 1614 | 1.48 | 1.31, 1.68 | <0.0001 | 1851 | 1.54 | 1.38, 1.73 | <0.0001 |
| Adjusted for age, sex, PCs, and max BMI | 1569 | 1.50 | 1.32, 1.71 | <0.0001 | 1798 | 1.57 | 1.39, 1.77 | <0.0001 |
| By ethnicity ^1^ |  |  |  |  |  |  |  |  |
| CHamoru | 716 | 1.36 | 1.15, 1.62 | 0.0003 | 851 | 1.40 | 1.19, 1.64 | <0.0001 |
| Filipino | 192 | 1.78 | 1.22, 2.60 | 0.0027 | 214 | 1.93 | 1.34, 2.77 | 0.0003 |
| Chuuk | 159 | 1.50 | 0.89, 2.53 | 0.1237 | 168 | 1.62 | 0.98, 2.66 | 0.0561 |
| Other | 547 | 1.64 | 1.30, 2.07 | <0.0001 | 618 | 1.71 | 1.38, 2.12 | <0.0001 |
| **Suzuki 2024 (AUC=0.798)** |  |  |  |  |  |  |  |  |
| Adjusted for age, sex, and PCs | 1614 | 1.59 | 1.40, 1.80 | <0.0001 | 1851 | 1.66 | 1.48, 1.86 | <0.0001 |
| Adjusted for age, sex, PCs, and max BMI | 1569 | 1.63 | 1.43, 1.86 | <0.0001 | 1798 | 1.72 | 1.52, 1.94 | <0.0001 |
| By ethnicity ^1^ |  |  |  |  |  |  |  |  |
| CHamoru | 716 | 1.51 | 1.27, 1.80 | <0.0001 | 851 | 1.60 | 1.36, 1.88 | <0.0001 |
| Filipino | 192 | 1.77 | 1.22, 2.55 | 0.0022 | 214 | 1.81 | 1.28, 2.56 | 0.0007 |
| Chuuk | 159 | 2.30 | 1.27, 4.16 | 0.0058 | 168 | 2.34 | 1.35, 4.03 | 0.0022 |
| Other | 547 | 1.57 | 1.24, 1.97 | 0.0001 | 618 | 1.63 | 1.32, 2.01 | <0.0001 |
| **DIAGRAM 2018 (AUC=0.787)** |  |  |  |  |  |  |  |  |
| Adjusted for age, sex, and PCs | 1614 | 1.34 | 1.19, 1.51 | <0.0001 | 1851 | 1.39 | 1.25, 1.55 | <0.0001 |
| Adjusted for age, sex, PCs, and max BMI | 1569 | 1.41 | 1.24, 1.60 | <0.0001 | 1798 | 1.46 | 1.30, 1.64 | <0.0001 |
| By ethnicity ^1^ |  |  |  |  |  |  |  |  |
| CHamoru | 716 | 1.23 | 1.04, 1.47 | 0.0159 | 851 | 1.33 | 1.14, 1.56 | 0.0003 |
| Filipino | 192 | 1.60 | 1.11, 2.28 | 0.0100 | 214 | 1.48 | 1.07, 2.05 | 0.0174 |
| Chuuk | 159 | 1.65 | 1.05, 2.57 | 0.0238 | 168 | 1.75 | 1.15, 2.67 | 0.0084 |
| Other | 547 | 1.36 | 1.09, 1.69 | 0.0056 | 618 | 1.37 | 1.12, 1.68 | 0.0021 |
| **DIAMANTE 2022 (AUC=0.794)** |  |  |  |  |  |  |  |  |
| Adjusted for age, sex, and PCs | 1614 | 1.51 | 1.34, 1.70 | <0.0001 | 1851 | 1.58 | 1.41, 1.77 | <0.0001 |
| Adjusted for age, sex, PCs, and max BMI | 1569 | 1.58 | 1.39, 1.80 | <0.0001 | 1798 | 1.66 | 1.47, 1.86 | <0.0001 |
| By ethnicity ^1^ |  |  |  |  |  |  |  |  |
| CHamoru | 716 | 1.51 | 1.27, 1.80 | <0.0001 | 851 | 1.58 | 1.35, 1.86 | <0.0001 |
| Filipino | 192 | 1.62 | 1.16, 2.27 | 0.0044 | 214 | 1.65 | 1.21, 2.24 | 0.0013 |
| Chuuk | 159 | 1.54 | 0.93, 2.56 | 0.0898 | 168 | 1.81 | 1.13, 2.90 | 0.0112 |
| Other | 547 | 1.45 | 1.16, 1.82 | 0.0010 | 618 | 1.52 | 1.24, 1.88 | <0.0001 |

^1^Adjusted for age, sex, and the first four genetic PCs.

# **Supplemental Table 4.** Associations of polygenic risk scores with type 2 diabetes (OR per SD) accounting for the genetic relatedness among individuals

|  | **OR (95% CI)** | **Pseudo R^2^** |
| --- | --- | --- |
| Khera 2018 | 1.48 (1.30, 1.68) | 0.020 |
| Ge 2022 | 1.76 (1.57, 1.97) | 0.052 |
| Mars 2022 | 1.81 (1.59, 2.06) | 0.045 |
| Prive 2022 | 1.52 (1.36, 1.70) | 0.030 |
| Suzuki 2024 | 1.65 (1.48, 1.84) | 0.043 |
| DIAGRAM 2018 | 1.39 (1.25, 1.55) | 0.020 |
| DIAMANTE 2022 | 1.59 (1.42, 1.78) | 0.038 |

^1^Adjusted for age, sex, first four genetic PCs, and accounting for genetic relatedness among individuals. Pseudo-R^2^ of polygenic scores by Nagelkerke’s method

# **Supplemental Table 5**. Associations of Polygenic Risk Scores derived from PRS-CSx (Ge, *Genome Medicine,* 2022), PRSice (Mars, *Am J Hum Gen*, 2022) and P&T (Suzuki, *Nature*, 2024) with Type 2 Diabetes (OR per SD) corrected for projected PCs derived from 1000 Genomes Project

|  | **n** | **OR** | **95% CI** | **p-value** |
| --- | --- | --- | --- | --- |
| **Ge 2022** |  |  |  |  |
| Adjusted for age, sex, and PCs | 1851 | 1.79 | 1.60, 2.01 | <0.0001 |
| Adjusted for age, sex, PCs, and max BMI | 1798 | 1.85 | 1.63, 2.09 | <0.0001 |
| By ethnicity ^1^ |  |  |  |  |
| CHamoru | 851 | 1.70 | 1.44, 2.01 | <0.0001 |
| Filipino | 214 | 2.06 | 1.47, 2.88 | <0.0001 |
| Chuuk | 168 | 2.00 | 1.24, 3.22 | 0.0040 |
| Other | 618 | 1.72 | 1.40, 2.12 | <0.0001 |
| **Mars 2022** |  |  |  |  |
| Adjusted for age, sex, and PCs | 1851 | 1.78 | 1.59, 1.99 | <0.0001 |
| Adjusted for age, sex, PCs, and max BMI | 1798 | 1.80 | 1.59, 2.03 | <0.0001 |
| By ethnicity ^1^ |  |  |  |  |
| CHamoru | 851 | 1.67 | 1.41, 1.96 | <0.0001 |
| Filipino | 214 | 1.91 | 1.36, 2.70 | 0.0002 |
| Chuuk | 168 | 1.97 | 1.17, 3.31 | 0.0100 |
| Other | 618 | 1.74 | 1.42, 2.13 | <0.0001 |
| **Suzuki 2024** |  |  |  |  |
| Adjusted for age, sex, and PCs | 1851 | 1.66 | 1.49, 1.86 | <0.0001 |
| Adjusted for age, sex, PCs, and max BMI | 1798 | 1.72 | 1.53, 1.94 | <0.0001 |
| By ethnicity ^1^ |  |  |  |  |
| CHamoru | 851 | 1.60 | 1.36, 1.87 | <0.0001 |
| Filipino | 214 | 1.85 | 1.31, 2.61 | 0.0005 |
| Chuuk | 168 | 2.37 | 1.38, 4.08 | 0.0016 |
| Other | 618 | 1.61 | 1.32, 1.96 | <0.0001 |

^1^Adjusted for age, sex, and PCs derived from the 1000 Genomes Project.

# **Supplemental Table 6.** Cochran’s test to examine differences by ethnicity in associations of polygenic risk scores with type 2 diabetes (OR per SD)

|  | **CHamoru** | **Filipino** | **Chuuk** | **Other** | **p-value** |
| --- | --- | --- | --- | --- | --- |
|  | **OR (95% CI)** | **OR (95% CI)** | **OR (95% CI)** | **OR (95% CI)** |  |
| Khera 2018 | 1.34 (1.12, 1.60) | 1.71 (1.16, 2.53) | 1.20 (0.66, 2.18) | 1.63 (1.29, 2.06) | 0.417 |
| Ge 2022 | 1.72 (1.45, 2.03) | 2.10 (1.50, 2.95) | 2.11 (1.29, 3.46) | 1.66 (1.34, 2.05) | 0.577 |
| Mars 2022 | 1.78 (1.47, 2.15) | 2.22 (1.50, 3.29) | 2.21 (1.20, 4.07) | 1.67 (1.32, 2.11) | 0.588 |
| Prive 2022 | 1.40 (1.19, 1.64) | 1.93 (1.34, 2.77) | 1.62 (0.98, 2.66) | 1.71 (1.38, 2.12) | 0.282 |
| Suzuki 2024 | 1.60 (1.36, 1.88) | 1.81 (1.28, 2.56) | 2.34 (1.35, 4.03) | 1.63 (1.32, 2.01) | 0.579 |
| DIAGRAM 2018 | 1.33 (1.14, 1.56) | 1.48 (1.07, 2.05) | 1.75 (1.15, 2.67) | 1.37 (1.12, 1.68) | 0.660 |
| DIAMANTE 2022 | 1.58 (1.35, 1.86) | 1.65 (1.21, 2.24) | 1.81 (1.13, 2.90) | 1.52 (1.24, 1.88) | 0.918 |

Estimates adjusted for age, sex, and the first four genetic PCs.

# **Supplemental Table 7.** Differences between associations of PRSs with T2D (OR per 5000 risk alleles) obtained in the Pacific Islander+Filipino population, and those obtained in European Americans from the ARIC study

|  | **GUAM** | **ARIC** | **P-HET** |
| --- | --- | --- | --- |
|  | **OR (95% CI)** | **OR (95% CI)** |  |
| Khera 2018^1^ | 1.50 (1.30, 1.72) | 2.39 (2.25, 2.52) | <0.001 |
| Ge 2022 | 2.19 (1.85, 2.58) | 2.34 (2.17, 2.52) | 0.483 |
| Mars 2022 | 2.11 (1.78, 2.49) | 2.32 (2.15, 2.50) | 0.308 |
| Prive 2022^2^ | 2.05 (1.70, 2.48) | 2.15 (1.98, 2.33) | 0.669 |
| Suzuki 2024^3^ | 1.96 (1.68, 2.28) | 2.23 (2.08, 2.39) | 0.132 |
| DIAGRAM 2018^4^ | 1.69 (1.42, 2.01) | 2.00 (1.85, 2.17) | 0.079 |
| DIAMANTE 2022^4^ | 1.67 (1.47, 1.89) | 1.78 (1.67, 1.89) | 0.354 |

^1^ OR per 15000 risk alleles.

^2^ OR per 500 risk alleles.

^3^ OR per 20 risk alleles.

^4^ OR per 10 risk alleles.

Note that European Americans from ARIC were included in the GWAS used to derive the Khera 2018 PRS, which, thus, may be subject “overfitting”.

# **Supplemental Table 8.** R^2^ and AUC differences between associations of PRSs with T2D obtained in the Pacific Islander+Filipino population, and those obtained in European Americans from the ARIC study

|  | **R^2^** | | **AUC ^a^** | |
| --- | --- | --- | --- | --- |
|  | **GUAM** | **ARIC** | **GUAM** | **ARIC** |
| Khera 2018 | 0.020 | 0.172 ^b^ | 0.579 | 0.708 |
| Ge 2022 | 0.055 | 0.092 | 0.620 | 0.655 |
| Mars 2022 | 0.047 | 0.095 | 0.604 | 0.664 |
| Prive 2022 | 0.033 | 0.059 | 0.588 | 0.631 |
| Suzuki 2024 | 0.047 | 0.087 | 0.606 | 0.660 |
| DIAGRAM 2018 | 0.021 | 0.052 | 0.588 | 0.623 |
| DIAMANTE 2022 | 0.039 | 0.062 | 0.603 | 0.635 |

^a^ Comparisons between GUAM and ARIC populations were performed using AUC models without covariates.

^b^ The relatively strong performance of the Khera PRS may be influenced by the inclusion of ARIC samples in its derivation, which could contribute to overfitting.

# **Supplemental Table 9.** Associations of T2D polygenic scores with fasting plasma glucose (mmol/L) per SD difference in PRS

|  | **Excluding participants with kidney failure** | | | | **Including participants with kidney failure** | | | |
| --- | --- | --- | --- | --- | --- | --- | --- | --- |
|  | **n** | **β** | **95% CI** | **p-value** | **n** | **β** | **95% CI** | **p-value** |
| **Khera 2018** |  |  |  |  |  |  |  |  |
| Adjusted for age, sex, and PCs | 1386 | 0.30 | 0.10, 0.49 | 0.0028 | 1414 | 0.30 | 0.11, 0.50 | 0.0022 |
| By ethnicity ^1^ |  |  |  |  |  |  |  |  |
| CHamoru | 590 | 0.12 | -0.17, 0.42 | 0.4160 | 607 | 0.14 | -0.15, 0.43 | 0.3514 |
| Filipino | 153 | 0.39 | -0.18, 0.96 | 0.1761 | 155 | 0.36 | -0.20, 0.92 | 0.2105 |
| Chuuk | 151 | 0.35 | -0.53, 1.23 | 0.4357 | 151 | 0.35 | -0.53, 1.22 | 0.4357 |
| Other | 492 | 0.48 | 0.17, 0.78 | 0.0020 | 501 | 0.47 | 0.17, 0.77 | 0.0020 |
| **Ge 2022** |  |  |  |  |  |  |  |  |
| Adjusted for age, sex, and PCs | 1386 | 0.46 | 0.28, 0.63 | <0.0001 | 1414 | 0.46 | 0.29, 0.63 | <0.0001 |
| By ethnicity ^1^ |  |  |  |  |  |  |  |  |
| CHamoru | 590 | 0.34 | 0.06, 0.61 | 0.0156 | 607 | 0.36 | 0.09, 0.63 | 0.0094 |
| Filipino | 153 | 0.20 | -0.27, 0.67 | 0.3951 | 155 | 0.18 | -0.28, 0.64 | 0.4387 |
| Chuuk | 151 | 0.57 | -0.08, 1.22 | 0.0855 | 151 | 0.57 | -0.08, 1.22 | 0.0855 |
| Other | 492 | 0.61 | 0.34, 0.89 | <0.0001 | 501 | 0.60 | 0.33, 0.87 | <0.0001 |
| **Mars 2022** |  |  |  |  |  |  |  |  |
| Adjusted for age, sex, and PCs | 1386 | 0.46 | 0.26, 0.66 | <0.0001 | 1414 | 0.47 | 0.27, 0.66 | <0.0001 |
| By ethnicity ^1^ |  |  |  |  |  |  |  |  |
| CHamoru | 590 | 0.37 | 0.05, 0.68 | 0.0227 | 607 | 0.39 | 0.08, 0.70 | 0.0139 |
| Filipino | 153 | 0.30 | -0.28, 0.89 | 0.3076 | 155 | 0.29 | -0.30, 0.87 | 0.3313 |
| Chuuk | 151 | 0.57 | -0.24, 1.39 | 0.1654 | 151 | 0.57 | -0.24, 1.39 | 0.1654 |
| Other | 492 | 0.54 | 0.24, 0.84 | 0.0005 | 501 | 0.54 | 0.24, 0.84 | 0.0005 |
| **Prive 2022** |  |  |  |  |  |  |  |  |
| Adjusted for age, sex, and PCs | 1386 | 0.35 | 0.18, 0.52 | <0.0001 | 1414 | 0.35 | 0.18, 0.52 | <0.0001 |
| By ethnicity ^1^ |  |  |  |  |  |  |  |  |
| CHamoru | 590 | 0.23 | -0.03, 0.49 | 0.0797 | 607 | 0.23 | -0.02, 0.49 | 0.0730 |
| Filipino | 153 | 0.32 | -0.19, 0.82 | 0.2127 | 155 | 0.31 | -0.19, 0.80 | 0.2283 |
| Chuuk | 151 | 0.22 | -0.40, 0.83 | 0.4843 | 151 | 0.22 | -0.40, 0.83 | 0.4843 |
| Other | 492 | 0.57 | 0.29, 0.86 | <0.0001 | 501 | 0.56 | 0.28, 0.84 | <0.0001 |
| **Suzuki 2024** |  |  |  |  |  |  |  |  |
| Adjusted for age, sex, and PCs | 1386 | 0.44 | 0.27, 0.61 | <0.0001 | 1414 | 0.44 | 0.27, 0.60 | <0.0001 |
| By ethnicity ^1^ |  |  |  |  |  |  |  |  |
| CHamoru | 590 | 0.26 | 0.01, 0.47 | 0.0455 | 607 | 0.28 | 0.03, 0.54 | 0.0305 |
| Filipino | 153 | 0.10 | -0.35, 0.56 | 0.6503 | 155 | 0.09 | -0.36, 0.54 | 0.6941 |
| Chuuk | 151 | 1.12 | 0.47, 1.77 | 0.0009 | 151 | 1.12 | 0.47, 1.77 | 0.0009 |
| Other | 492 | 0.58 | 0.30, 0.85 | <0.0001 | 501 | 0.57 | 0.30, 0.84 | <.0001 |
| **DIAGRAM 2018** |  |  |  |  |  |  |  |  |
| Adjusted for age, sex, and PCs | 1386 | 0.30 | 0.13, 0.47 | 0.0004 | 1414 | 0.31 | 0.14, 0.48 | 0.0003 |
| By ethnicity ^1^ |  |  |  |  |  |  |  |  |
| CHamoru | 590 | 0.11 | -0.16, 0.37 | 0.4224 | 607 | 0.12 | -0.14, 0.39 | 0.3590 |
| Filipino | 153 | 0.02 | -0.46, 0.49 | 0.9504 | 155 | 0.01 | -0.47, 0.48 | 0.9815 |
| Chuuk | 151 | 0.63 | 0.08, 1.18 | 0.0256 | 151 | 0.63 | 0.08, 1.18 | 0.0256 |
| Other | 492 | 0.48 | 0.20, 0.75 | 0.0006 | 501 | 0.48 | 0.21, 0.74 | 0.0005 |
| **DIAMANTE 2022** |  |  |  |  |  |  |  |  |
| Adjusted for age, sex, and PCs | 1386 | 0.42 | 0.26, 0.59 | <0.0001 | 1414 | 0.42 | 0.25, 0.58 | <0.0001 |
| By ethnicity ^1^ |  |  |  |  |  |  |  |  |
| CHamoru | 590 | 0.33 | 0.08, 0.58 | 0.0111 | 607 | 0.35 | 0.10, 0.60 | 0.0057 |
| Filipino | 153 | -0.24 | -0.71, 0.23 | 0.3176 | 155 | -0.25 | -0.71, 0.21 | 0.2820 |
| Chuuk | 151 | 0.98 | 0.38, 1.59 | 0.0017 | 151 | 0.98 | 0.38, 1.59 | 0.0017 |
| Other | 492 | 0.55 | 0.27, 0.83 | 0.0001 | 501 | 0.53 | 0.25, 0.80 | 0.0002 |

^1^Adjusted for age, sex, and PCs

# **Supplemental Table 10.** Associations of T2D polygenic scores with HbA1c (mmol/mol) per SD difference in PRS

|  | **Excluding participants with kidney failure** | | | | **Including participants with kidney failure** | | | |
| --- | --- | --- | --- | --- | --- | --- | --- | --- |
|  | **n** | **β** | **95% CI** | **p-value** | **n** | **β** | **95% CI** | **p-value** |
| **Khera 2018** |  |  |  |  |  |  |  |  |
| Adjusted for age, sex, and PCs | 1624 | 2.98 | 1.76, 4.20 | <0.0001 | 1641 | 2.87 | 1.65, 4.09 | <0.0001 |
| By ethnicity ^1^ |  |  |  |  |  |  |  |  |
| CHamoru | 723 | 3.04 | 1.19, 4.88 | 0.0013 | 727 | 2.95 | 1.10, 4.80 | 0.0018 |
| Filipino | 196 | 1.95 | -1.86, 5.77 | 0.3142 | 197 | 1.95 | -1.85, 5.76 | 0.3130 |
| Chuuk | 160 | 1.73 | -2.55, 6.02 | 0.3879 | 160 | 1.73 | -2.55, 6.02 | 0.4262 |
| Other | 545 | 3.61 | 1.59, 5.64 | 0.0005 | 557 | 3.37 | 1.38, 5.37 | 0.0009 |
| **Ge 2022** |  |  |  |  |  |  |  |  |
| Adjusted for age, sex, and PCs | 1624 | 4.46 | 3.40, 5.53 | <0.0001 | 1641 | 4.32 | 3.26, 5.38 | <0.0001 |
| By ethnicity ^1^ |  |  |  |  |  |  |  |  |
| CHamoru | 723 | 4.91 | 3.25, 6.57 | <0.0001 | 727 | 4.93 | 3.27, 6.58 | <0.0001 |
| Filipino | 196 | 5.10 | 2.08, 8.11 | 0.0010 | 197 | 5.09 | 2.09, 8.10 | 0.0010 |
| Chuuk | 160 | 3.34 | 0.11, 6.57 | 0.0428 | 160 | 3.34 | 0.11, 6.57 | 0.0428 |
| Other | 545 | 4.06 | 2.24, 5.88 | <0.0001 | 557 | 3.60 | 1.80, 5.39 | <0.0001 |
| **Mars 2022** |  |  |  |  |  |  |  |  |
| Adjusted for age, sex, and PCs | 1624 | 4.76 | 3.54, 5.99 | <0.0001 | 1641 | 4.63 | 3.42, 5.84 | <0.0001 |
| By ethnicity ^1^ |  |  |  |  |  |  |  |  |
| CHamoru | 723 | 5.38 | 3.47, 7.28 | <0.0001 | 727 | 5.39 | 3.49, 7.29 | <0.0001 |
| Filipino | 196 | 5.56 | 1.90, 9.22 | 0.0031 | 197 | 5.54 | 1.90, 9.18 | 0.0031 |
| Chuuk | 160 | 3.71 | -0.25, 7.68 | 0.0663 | 160 | 3.71 | -0.25, 7.68 | 0.0663 |
| Other | 545 | 4.03 | 2.03, 6.03 | <0.0001 | 557 | 3.64 | 1.67, 5.62 | 0.0003 |
| **Prive 2022** |  |  |  |  |  |  |  |  |
| Adjusted for age, sex, PCs | 1624 | 3.25 | 2.18, 4.33 | <0.0001 | 1641 | 3.23 | 2.16, 4.30 | <0.0001 |
| By ethnicity ^1^ |  |  |  |  |  |  |  |  |
| CHamoru | 723 | 2.64 | 1.01, 4.27 | 0.0015 | 727 | 2.68 | 1.05, 4.31 | 0.0013 |
| Filipino | 196 | 6.23 | 2.98, 9.49 | 0.0002 | 197 | 6.18 | 2.95, 9.42 | 0.0002 |
| Chuuk | 160 | 1.04 | -2.02, 4.11 | 0.5017 | 160 | 1.04 | -2.02, 4.11 | 0.5017 |
| Other | 545 | 3.98 | 2.11, 5.84 | <0.0001 | 557 | 3.87 | 2.04, 5.70 | <0.0001 |
| **Suzuki 2024** |  |  |  |  |  |  |  |  |
| Adjusted for age, sex, and PCs | 1624 | 4.29 | 3.24, 5.34 | <0.0001 | 1641 | 4.20 | 3.15, 5.25 | <0.0001 |
| By ethnicity ^1^ |  |  |  |  |  |  |  |  |
| CHamoru | 723 | 4.76 | 3.17, 6.35 | <0.0001 | 727 | 4.72 | 3.14, 6.31 | <0.0001 |
| Filipino | 196 | 3.12 | 0.01, 6.23 | 0.0492 | 197 | 3.12 | 0.02, 6.22 | 0.0485 |
| Chuuk | 160 | 5.94 | 2.70, 9.18 | 0.0004 | 160 | 5.94 | 2.70, 9.18 | 0.0004 |
| Other | 545 | 3.62 | 1.82, 5.43 | 0.0001 | 557 | 3.41 | 1.62, 5.19 | 0.0002 |
| **DIAGRAM 2018** |  |  |  |  |  |  |  |  |
| Adjusted for age, sex, and PCs | 1624 | 2.69 | 1.63, 3.76 | <0.0001 | 1641 | 2.68 | 1.62, 3.74 | <0.0001 |
| By ethnicity ^1^ |  |  |  |  |  |  |  |  |
| CHamoru | 723 | 2.46 | 0.78, 4.13 | 0.0040 | 727 | 2.58 | 0.91, 4.26 | 0.0025 |
| Filipino | 196 | 3.34 | 0.18, 6.51 | 0.0381 | 197 | 3.29 | 0.17, 6.41 | 0.0388 |
| Chuuk | 160 | 3.00 | 0.26, 5.73 | 0.0319 | 160 | 3.00 | 0.26, 5.73 | 0.0319 |
| Other | 545 | 2.56 | 0.74, 4.39 | 0.0058 | 557 | 2.38 | 0.58, 4.18 | 0.0096 |
| **DIAMANTE 2022** |  |  |  |  |  |  |  |  |
| Adjusted for age, sex, and PCs | 1624 | 4.04 | 3.00, 5.08 | <0.0001 | 1641 | 3.95 | 2.92, 4.99 | <0.0001 |
| By ethnicity ^1^ |  |  |  |  |  |  |  |  |
| CHamoru | 723 | 4.52 | 2.95, 6.09 | <0.0001 | 727 | 4.57 | 3.00, 6.14 | <0.0001 |
| Filipino | 196 | 3.08 | 0.07, 6.10 | 0.0449 | 197 | 3.06 | 0.07, 6.06 | 0.0447 |
| Chuuk | 160 | 4.94 | 1.96, 7.92 | 0.0013 | 160 | 4.94 | 1.96, 7.92 | 0.0013 |
| Other | 545 | 3.31 | 1.48, 5.15 | 0.0004 | 557 | 3.04 | 1.23, 4.84 | 0.0010 |

^1^Adjusted for age, sex, and PCs

# **Supplemental Table 11.** Associations of T2D polygenic scores with fasting plasma glucose and HbA1c in participants without diabetes

|  | **Fasting plasma glucose, mmol/L**  **n=1105** | | **HbA1c, mmol/mol**  **n=1115** | |
| --- | --- | --- | --- | --- |
|  | **β (95% CI)** | **p-value** | **β (95% CI)** | **p-value** |
| Khera 2018 | 0.011(-0.027, 0.049) | 0.572 | 0.23 (-0.01, 0.48) | 0.060 |
| Ge 2022 | 0.028 (-0.007, 0.063) | 0.123 | 0.29 (0.06, 0.51) | 0.011 |
| Mars 2022 | 0.022 (-0.018, 0.063) | 0.277 | 0.33 (0.08, 0.59) | 0.010 |
| Prive 2022 | 0.001 (-0.034, 0.035) | 0.983 | 0.33 (0.11, 0.55) | 0.002 |
| Suzuki 2024 | 0.031 (-0.003, 0.065) | 0.074 | 0.37 (0.16, 0.59) | <0.001 |
| DIAGRAM 2018 | 0.005 (-0.028, 0.039) | 0.745 | 0.17 (-0.04, 0.38) | 0.119 |
| DIAMANTE 2022 | 0.031 (-0.002, 0.065) | 0.066 | 0.33 (0.11, 0.54) | 0.002 |

Adjusted for age, sex, and PCs

# **Supplemental Table 12.** Associations of T2D polygenic scores with maximum BMI (kg/m^2^) per SD difference in PRS

|  | **Excluding participants with kidney failure** | | | | **Including participants with kidney failure** | | | |
| --- | --- | --- | --- | --- | --- | --- | --- | --- |
|  | **n** | **β** | **95% CI** | **p-value** | **n** | **β** | **95% CI** | **p-value** |
| **Khera 2018** |  |  |  |  |  |  |  |  |
| Adjusted for age, sex, and PCs | 1617 | 0.25 | -0.20, 0.70 | 0.2743 | 1933 | 0.19 | -0.23, 0.62 | 0.3791 |
| By ethnicity ^1^ |  |  |  |  |  |  |  |  |
| CHamoru | 721 | 0.28 | -0.43, 1.01 | 0.4354 | 882 | 0.05 | -0.62, 0.74 | 0.8643 |
| Filipino | 195 | -0.01 | -0.84, 0.81 | 0.9654 | 237 | 0.28 | -0.53, 1.09 | 0.4970 |
| Chuuk | 145 | 1.23 | -0.33, 2.81 | 0.1229 | 156 | 1.75 | 0.28, 3.21 | 0.0196 |
| Other | 556 | 0.06 | -0.69, 0.83 | 0.8607 | 658 | 0.01 | -0.70, 0.73 | 0.9646 |
| **Ge 2022** |  |  |  |  |  |  |  |  |
| Adjusted for age, sex, and PCs | 1617 | 0.23 | -0.17. 0.63 | 0.2635 | 1933 | 0.21 | -0.16, 0.59 | 0.2690 |
| By ethnicity ^1^ |  |  |  |  |  |  |  |  |
| CHamoru | 721 | 0.37 | -0.29, 1.03 | 0.2701 | 882 | 0.12 | -0.49, 0.75 | 0.6843 |
| Filipino | 195 | -0.25 | -0.92, 0.42 | 0.4649 | 237 | 0.32 | -0.34, 1.00 | 0.3403 |
| Chuuk | 145 | 0.11 | -1.10, 1.33 | 0.8574 | 156 | 0.15 | -1.02, 1.32 | 0.7961 |
| Other | 556 | 0.12 | -0.57, 0.82 | 0.7258 | 658 | 0.16 | -0.47, 0.81 | 0.6120 |
| **Mars 2022** |  |  |  |  |  |  |  |  |
| Adjusted for age, sex, and PCs | 1617 | 0.31 | -0.14, 0.76 | 0.1846 | 1933 | 0.26 | -0.16, 0.70 | 0.2232 |
| By ethnicity ^1^ |  |  |  |  |  |  |  |  |
| CHamoru | 721 | 0.57 | -0.17, 1.33 | 0.1345 | 882 | 0.35 | -0.35, 1.05 | 0.3276 |
| Filipino | 195 | -0.31 | -1.13, 0.49 | 0.4374 | 237 | 0.12 | -0.69, 0.93 | 0.7725 |
| Chuuk | 145 | -0.00 | -1.49, 1.49 | 0.9968 | 156 | 0.07 | -1.36, 1.51 | 0.9217 |
| Other | 556 | 0.08 | -0.67, 0.85 | 0.7569 | 658 | 0.06 | -0.65, 0.77 | 0.8713 |
| **Prive 2022** |  |  |  |  |  |  |  |  |
| Adjusted for age, sex, and PCs | 1617 | 0.19 | -0.20, 0.59 | 0.3446 | 1933 | 0.24 | -0.14, 0.62 | 0.2183 |
| By ethnicity ^1^ |  |  |  |  |  |  |  |  |
| CHamoru | 721 | 0.41 | -0.22, 1.05 | 0.2617 | 882 | 0.41 | -0.19, 1.01 | 0.1790 |
| Filipino | 195 | 0.30 | -0.42, 1.04 | 0.4102 | 237 | 0.87 | 0.12, 1.61 | 0.0222 |
| Chuuk | 145 | -0.95 | -2.06, 0.16 | 0.0934 | 156 | -1.05 | -2.14, 0.03 | 0.0587 |
| Other | 556 | 0.14 | -0.57, 0.86 | 0.6927 | 658 | -0.02 | -0.69, 0.64 | 0.9486 |
| **Suzuki 2024** |  |  |  |  |  |  |  |  |
| Adjusted for age, sex, and PCs | 1617 | 0.14 | -0.25, 0.54 | 0.4818 | 1933 | 0.09 | -0.27, 0.47 | 0.6081 |
| By ethnicity ^1^ |  |  |  |  |  |  |  |  |
| CHamoru | 721 | 0.28 | -0.35, 0.92 | 0.3875 | 882 | 0.06 | -0.53, 0.66 | 0.8353 |
| Filipino | 195 | -0.62 | -1.32, 0.07 | 0.0778 | 237 | -0.09 | -0.78, 0.59 | 0.7862 |
| Chuuk | 145 | 0.38 | -0.87, 1.65 | 0.5420 | 156 | 0.31 | -0.89, 1.52 | 0.6035 |
| Other | 556 | 0.14 | -0.54, 0.83 | 0.6827 | 658 | 0.10 | -0.52, 0.73 | 0.7379 |
| **DIAGRAM 2018** |  |  |  |  |  |  |  |  |
| Adjusted for age, sex, and PCs | 1617 | -0.14 | -0.54, 0.25 | 0.4795 | 1933 | -0.06 | -0.43, 0.31 | 0.7446 |
| By ethnicity ^1^ |  |  |  |  |  |  |  |  |
| CHamoru | 721 | -0.24 | -0.90, 0.41 | 0.4675 | 882 | -0.16 | -0.77, 0.44 | 0.6012 |
| Filipino | 195 | -0.79 | -1.48, -0.09 | 0.0264 | 237 | -0.45 | -1.14, 0.22 | 0.1861 |
| Chuuk | 145 | 0.05 | -1.01, 1.13 | 0.9143 | 156 | 0.20 | -0.83, 1.24 | 0.6945 |
| Other | 556 | 0.01 | -0.67, 0.69 | 0.9704 | 658 | -0.00 | -0.65, 0.64 | 0.9848 |
| **DIAMANTE 2022** |  |  |  |  |  |  |  |  |
| Adjusted for age, sex, and PCs | 1617 | -0.05 | -0.44, 0.34 | 0.8020 | 1933 | -0.02 | -0.39, 0.34 | 0.8881 |
| By ethnicity ^1^ |  |  |  |  |  |  |  |  |
| CHamoru | 721 | -0.08 | -0.71, 0.54 | 0.8008 | 882 | -0.18 | -0.77, 0.41 | 0.5468 |
| Filipino | 195 | -0.78 | -1.45, -0.12 | 0.0204 | 237 | -0.20 | -0.85, 0.44 | 0.5283 |
| Chuuk | 145 | -0.17 | -1.37, 1.02 | 0.9286 | 156 | -0.20 | -1.36, 0.95 | 0.7294 |
| Other | 556 | 0.23 | -0.46, 0.93 | 0.5065 | 658 | 0.19 | -0.45, 0.83 | 0.5608 |

^1^Adjusted for age, sex, and PCs.

# **Supplemental Table 13.** Associations of T2D polygenic scores with HOMA-IR (μIU/mL*mmol/L) per SD difference in PRS in participants without diabetes

|  | **n** | **β** | **95% CI** | **p-value** |
| --- | --- | --- | --- | --- |
| **Khera 2018** |  |  |  |  |
| Adjusted for age, sex, and PCs | 989 | 1.01 | 0.95, 1.07 | 0.6630 |
| By ethnicity ^1^ |  |  |  |  |
| CHamoru | 435 | 1.00 | 0.91, 1.10 | 0.9204 |
| Filipino | 99 | 0.97 | 0.79, 1.19 | 0.7927 |
| Chuuk | 100 | 1.15 | 0.88, 1.49 | 0.2954 |
| Other | 355 | 0.98 | 0.88, 1.09 | 0.7966 |
| **Ge 2022** |  |  |  |  |
| Adjusted for age, sex, and PCs | 989 | 1.01 | 0.95, 1.07 | 0.6111 |
| By ethnicity ^1^ |  |  |  |  |
| CHamoru | 435 | 1.02 | 0.93, 1.11 | 0.6841 |
| Filipino | 99 | 0.88 | 0.74, 1.05 | 0.1536 |
| Chuuk | 100 | 1.14 | 0.92, 1.41 | 0.2177 |
| Other | 355 | 1.01 | 0.91, 1.11 | 0.9021 |
| **Mars 2022** |  |  |  |  |
| Adjusted for age, sex, and PCs | 989 | 1.03 | 0.97, 1.10 | 0.3188 |
| By ethnicity ^1^ |  |  |  |  |
| CHamoru | 435 | 1.04 | 0.95, 1.17 | 0.3036 |
| Filipino | 99 | 0.85 | 0.69, 1.06 | 0.1456 |
| Chuuk | 100 | 1.09 | 0.84, 1.41 | 0.5262 |
| Other | 355 | 1.03 | 0.93, 1.14 | 0.5642 |
| **Prive 2022** |  |  |  |  |
| Adjusted for age, sex, PCs | 989 | 1.03 | 0.98, 1.09 | 0.2587 |
| By ethnicity ^1^ |  |  |  |  |
| CHamoru | 435 | 1.05 | 0.97, 1.14 | 0.2045 |
| Filipino | 99 | 1.00 | 0.82, 1.21 | 0.9696 |
| Chuuk | 100 | 0.92 | 0.76, 1.10 | 0.3511 |
| Other | 355 | 1.04 | 0.94, 1.14 | 0.4785 |
| **Suzuki 2024** |  |  |  |  |
| Adjusted for age, sex, and PCs | 989 | 1.02 | 0.96, 1.07 | 0.5686 |
| By ethnicity ^1^ |  |  |  |  |
| CHamoru | 435 | 0.99 | 0.91, 1.08 | 0.8468 |
| Filipino | 99 | 0.93 | 0.79, 1.10 | 0.3881 |
| Chuuk | 100 | 1.01 | 0.81, 1.27 | 0.9070 |
| Other | 355 | 1.08 | 0.98, 1.18 | 0.1359 |
| **DIAGRAM 2018** |  |  |  |  |
| Adjusted for age, sex, and PCs | 989 | 0.98 | 0.93, 1.03 | 0.4282 |
| By ethnicity ^1^ |  |  |  |  |
| CHamoru | 435 | 0.96 | 0.89, 1.05 | 0.3948 |
| Filipino | 99 | 0.92 | 0.78, 1.09 | 0.3493 |
| Chuuk | 100 | 0.97 | 0.81, 1.17 | 0.7786 |
| Other | 355 | 1.01 | 0.92, 1.11 | 0.8075 |
| **DIAMANTE 2022** |  |  |  |  |
| Adjusted for age, sex, and PCs | 989 | 1.00 | 0.95, 1.06 | 0.9187 |
| By ethnicity ^1^ |  |  |  |  |
| CHamoru | 435 | 1.00 | 0.93, 1.09 | 0.9197 |
| Filipino | 99 | 0.99 | 0.82, 1.18 | 0.8885 |
| Chuuk | 100 | 0.98 | 0.80, 1.19 | 0.8234 |
| Other | 355 | 1.00 | 0.91, 1.10 | 0.9817 |

^1^Adjusted for age, sex, and PCs

# **Supplemental Table 14.** Association of T2D polygenic score with HOMA-B (μIU/mL) per SD difference in PRS in participants without diabetes

|  | **n** | **β** | **95% CI** | **p-value** |
| --- | --- | --- | --- | --- |
| **Khera 2018** |  |  |  |  |
| Adjusted for age, sex, and PCs | 988 | 1.00 | 0.94, 1.06 | 0.8983 |
| By ethnicity ^1^ |  |  |  |  |
| CHamoru | 434 | 1.01 | 0.93, 1.10 | 0.7560 |
| Filipino | 99 | 0.96 | 0.79, 1.16 | 0.6761 |
| Chuuk | 100 | 1.14 | 0.90, 1.45 | 0.2568 |
| Other | 355 | 0.96 | 0.88, 1.06 | 0.4914 |
| **Ge 2022** |  |  |  |  |
| Adjusted for age, sex, and PCs | 988 | 0.98 | 0.93, 1.03 | 0.4780 |
| By ethnicity ^1^ |  |  |  |  |
| CHamoru | 434 | 0.98 | 0.90, 1.06 | 0.5946 |
| Filipino | 99 | 0.86 | 0.73, 1.01 | 0.0698 |
| Chuuk | 100 | 1.12 | 0.92, 1.35 | 0.2536 |
| Other | 355 | 0.98 | 0.90, 1.07 | 0.6454 |
| **Mars 2022** |  |  |  |  |
| Adjusted for age, sex, and PCs | 988 | 1.01 | 0.95, 1.07 | 0.8316 |
| By ethnicity ^1^ |  |  |  |  |
| CHamoru | 434 | 1.03 | 0.94, 1.13 | 0.5496 |
| Filipino | 99 | 0.83 | 0.68, 1.02 | 0.0724 |
| Chuuk | 100 | 1.07 | 0.84, 1.35 | 0.5803 |
| Other | 355 | 1.00 | 0.91, 1.10 | 0.9322 |
| **Prive 2022** |  |  |  |  |
| Adjusted for age, sex, PCs | 988 | 1.02 | 0.97, 1.07 | 0.5433 |
| By ethnicity ^1^ |  |  |  |  |
| CHamoru | 434 | 1.04 | 0.97, 1.12 | 0.2586 |
| Filipino | 99 | 1.02 | 0.85, 1.22 | 0.8218 |
| Chuuk | 100 | 0.89 | 0.75, 1.05 | 0.1581 |
| Other | 355 | 1.01 | 0.93, 1.11 | 0.7925 |
| **Suzuki 2024** |  |  |  |  |
| Adjusted for age, sex, and PCs | 988 | 0.98 | 0.93, 1.03 | 0.4679 |
| By ethnicity ^1^ |  |  |  |  |
| CHamoru | 434 | 0.95 | 0.88, 1.02 | 0.1811 |
| Filipino | 99 | 0.91 | 0.78, 1.06 | 0.2110 |
| Chuuk | 100 | 0.99 | 0.81, 1.21 | 0.9395 |
| Other | 355 | 1.04 | 0.96, 1.14 | 0.3242 |
| **DIAGRAM 2018** |  |  |  |  |
| Adjusted for age, sex, and PCs | 988 | 0.97 | 0.92, 1.02 | 0.2444 |
| By ethnicity ^1^ |  |  |  |  |
| CHamoru | 434 | 0.95 | 0.88, 1.02 | 0.1664 |
| Filipino | 99 | 0.92 | 0.79, 1.07 | 0.2910 |
| Chuuk | 100 | 0.98 | 0.83, 1.15 | 0.7871 |
| Other | 355 | 1.01 | 0.93, 1.10 | 0.8201 |
| **DIAMANTE 2022** |  |  |  |  |
| Adjusted for age, sex, and PCs | 988 | 0.97 | 0.92, 1.02 | 0.1798 |
| By ethnicity ^1^ |  |  |  |  |
| CHamoru | 434 | 0.97 | 0.90, 1.04 | 0.3822 |
| Filipino | 99 | 0.94 | 0.80, 1.12 | 0.5036 |
| Chuuk | 100 | 1.02 | 0.85, 1.22 | 0.8657 |
| Other | 355 | 0.95 | 0.87, 1.04 | 0.2715 |

^1^Adjusted for age, sex, and PCs

# **Supplemental Table 15.** Associations of Polygenic Risk Scores derived from PRS-CSx (Ge, *Genome Medicine,* 2022), PRSice (Mars, *Am J Hum Gen*, 2022) and P&T (Suzuki, *Nature*, 2024) with clinical measures (per SD difference in PRS) corrected for projected PCs derived from 1000 Genomes Project

|  | **Maximum BMI**  **n=1933** | | | **Fasting plasma glucose, mmol/L**  **n=1414** | | | **HbA1C, mmol/mol**  **n=1641** | | | **HOMA-IR**  **n=989** | | | **HOMA- β**  **n=988** | | |
| --- | --- | --- | --- | --- | --- | --- | --- | --- | --- | --- | --- | --- | --- | --- | --- |
|  | **β (95% CI)** | **p-value** | **β (95% CI)** | | **p-value** | **β (95% CI)** | | **p-value** | **β (95% CI)** | | **p-value** | **β (95% CI)** | | **p-value** |  |
| **Ge 2022** |  |  |  | |  |  | |  |  | |  |  | |  |  |
| Adjusted for age, sex, and PCs | 0.46 (0.08, 0.84) | 0.0160 | 0.49 (0.32, 0.66) | | <0.0001 | 4.61 (3.57, 5.65) | | <0.0001 | 1.03 (0.97, 1.09) | | 0.3177 | 0.99 (0.94, 1.04) | | 0.7648 |  |
| By ethnicity ^1^ |  |  |  | |  |  | |  |  | |  |  | |  |  |
| CHamoru | 0.21 (-0.39, 0.82) | 0.4959 | 0.35 (0.08, 0.62) | | 0.0107 | 4.95 (3.33, 6.58) | | <0.0001 | 1.01 (0.93, 1.11) | | 0.6956 | 0.98 (0.90, 1.06) | | 0.5725 |  |
| Filipino | 0.30 (-0.36, 0.97) | 0.3646 | 0.15 (-0.29, 0.59) | | 0.5007 | 4.81 (1.88, 7.75) | | 0.0014 | 0.90 (0.76, 1.07) | | 0.2586 | 0.88 (0.75, 1.04) | | 0.1245 |  |
| Chuuk | 0.06 (-1.06, 1.19) | 0.9096 | 0.54 (-0.11, 1.17) | | 0.1076 | 3.07 (-0.11, 6.25) | | 0.0584 | 1.13 (0.93, 1.38) | | 0.2083 | 1.11 (0.93, 1.33) | | 0.2587 |  |
| Other | 0.36 (-0.27, 1.00) | 0.2668 | 0.63 (0.37, 0.90) | | <0.0001 | 4.00 (2.25, 5.76) | | <0.0001 | 1.00 (0.91, 1.10) | | 0.8828 | 0.99 (0.91, 1.07) | | 0.7461 |  |
| **Mars 2022** |  |  |  | |  |  | |  |  | |  |  | |  |  |
| Adjusted for age, sex, and PCs | 0.63 (0.25, 1.01) | 0.0010 | 0.47 (0.30, 0.64) | | <0.0001 | 4.59 (3.56, 5.63) | | <0.0001 | 1.05 (0.99, 1.11) | | 0.0768 | 1.02 (0.96, 1.07) | | 0.4338 |  |
| By ethnicity ^1^ |  |  |  | |  |  | |  |  | |  |  | |  |  |
| CHamoru | 0.43 (-0.17, 1.04) | 0.1594 | 0.33 (0.06, 0.60) | | 0.0154 | 4.86 (3.23, 6.49) | | <0.0001 | 1.05 (0.96, 1.15) | | 0.2187 | 1.02 (0.95, 1.11) | | 0.4769 |  |
| Filipino | 0.02 (-0.69, 0.74) | 0.9428 | 0.19 (-0.29, 0.68) | | 0.4344 | 4.40 (1.20, 7.59) | | 0.0071 | 0.90 (0.75, 1.09) | | 0.2924 | 0.88 (0.73, 1.05) | | 0.1601 |  |
| Chuuk | 0.01 (-1.17, 1.19) | 0.9904 | 0.55 (-0.14, 1.24) | | 0.1216 | 3.31 (-0.08, 6.71) | | 0.0562 | 1.04 (0.84, 1.30) | | 0.6653 | 1.03 (0.84, 1.25) | | 0.7722 |  |
| Other | 0.33 (-0.30, 0.96) | 0.3085 | 0.55 (0.29, 0.81) | | <0.0001 | 3.98 (2.26, 5.69) | | <0.0001 | 1.03 (0.93, 1.13) | | 0.5345 | 1.01 (0.93, 1.10) | | 0.7795 |  |
| **Suzuki 2024** |  |  |  | |  |  | |  |  | |  |  | |  |  |
| Adjusted for age, sex, and PCs | 0.19 (-0.18. 0.57) | 0.3076 | 0.44 (0.28, 0.61) | | <0.0001 | 4.29 (3.25, 5.34) | | <0.0001 | 1.02 (0.96, 1.07) | | 0.4830 | 0.98 (0.93, 1.03) | | 0.5686 |  |
| By ethnicity ^1^ |  |  |  | |  |  | |  |  | |  |  | |  |  |
| CHamoru | 0.10 (-0.48, 0.70) | 0.7190 | 0.26 (0.01, 0.52) | | 0.0372 | 4.73 (3.15, 6.30) | | <0.0001 | 0.99 (0.91, 1.07) | | 0.8969 | 0.95 (0.88, 1.02) | | 0.1912 |  |
| Filipino | -0.08 (-0.76, 0.59) | 0.8148 | 0.12 (-0.30, 0.55) | | 0.5683 | 3.15 (0.11, 6.19) | | 0.0423 | 0.92 (0.78, 1.08) | | 0.3243 | 0.89 (0.77, 1.04) | | 0.1614 |  |
| Chuuk | 0.12 (-1.07, 1.32) | 0.8372 | 1.14 (0.48, 1.79) | | 0.0007 | 6.08 (2.82, 9.34) | | 0.0003 | 1.00 (0.80, 1.24) | | 0.9780 | 0.97 (0.80, 1.19) | | 0.8324 |  |
| Other | 0.25 (-0.37, 0.88) | 0.4287 | 0.59 (0.32, 0.86) | | <0.0001 | 3.59 (1.82, 5.37) | | <0.0001 | 1.07 (0.97, 1.18) | | 0.1450 | 1.04 (0.96, 1.14) | | 0.2979 |  |

^1^Adjusted for age, sex, and PCs derived from the 1000 Genomes Project.


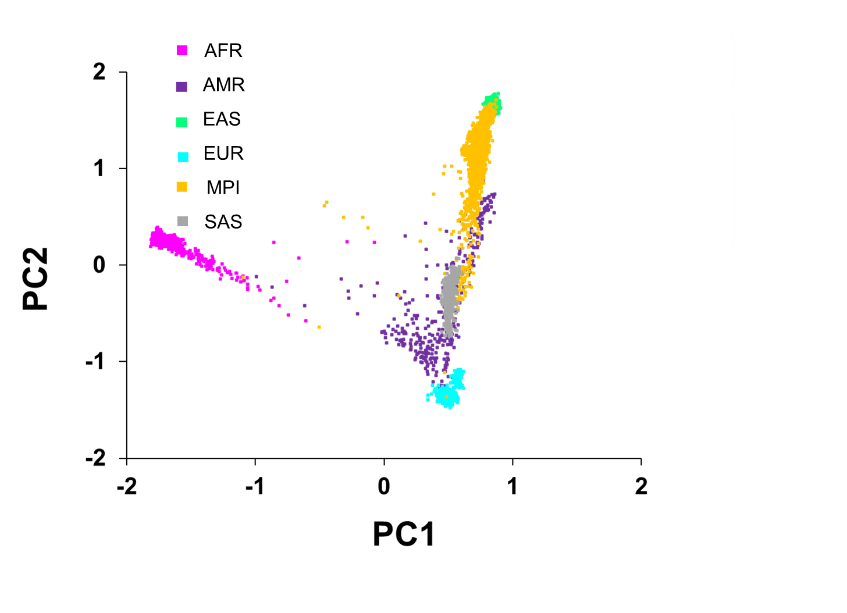


# **Supplemental Figure 1.** Principal Component Analysis. PC1 versus PC2 derived from the 1000 Genomes project, projected into the current study samples**.**

Abbreviations: AFR African, AMR Admixed American, EAS East Asian, EUR European, MPI Mariana Pacific Islander, and SAS South Asians.

V_ST_=0.010 (0.003, 0.016)

V_ST_=0.709 (0.693, 0.725)

# **Supplemental Figure 2.** Distribution of Mars 2022 PRS. Data represent the mean value (±1 SD) of the PRS for each population. VST is the phenotypic differentiation coefficient (with 95% confidence intervals).

Abbreviations: AFR African, AMR Admixed American, EAS East Asian, EUR European, MPI Mariana Pacific Islander, and SAS South Asians.

**Supplemental Figure 3**. Distribution of Suzuki 2024 PRS. Data represent the mean value (±1 SD) of the PRS for each population. VST is the phenotypic differentiation coefficient (with 95% confidence intervals).

V_ST_=0.001 (-0.002, 0.003)

V_ST_=0.306 (0.282, 0.329)

Unadjusted

Adj.-1000G-PCs

Abbreviations: AFR African, AMR Admixed American, EAS East Asian, EUR European, MPI Mariana Pacific Islander, and SAS South Asians.

Adj.-1000G-PCs

Unadjusted

V_ST_=0.016 (0.008, 0.024)

V_ST_=0.144 (0.126, 0.163)

# **Supplemental Figure 4.** Distribution of DIAMANTE 2022 PRS. Data represent the mean value (±1 SD) of the PRS for each population. VST is the phenotypic differentiation coefficient (with 95% confidence intervals).

Abbreviations: AFR African, AMR Admixed American, EAS East Asian, EUR European, MPI Mariana Pacific Islander, and SAS South Asians.

V_ST_=0.001 (-0.003, 0.001)

V_ST_=0.103 (0.085, 0.121)

Unadjusted

Adj.-1000G-PCs

# **Supplemental Figure 5.** Distribution of Prive 2022 PRS. Data represent the mean value (±1 SD) of the PRS for each population. VST is the phenotypic differentiation coefficient (with 95% confidence intervals).

Abbreviations: AFR African, AMR Admixed American, EAS East Asian, EUR European, MPI Mariana Pacific Islander, and SAS South Asians.

**Supplemental Figure 6**. Distribution of Khera 2018 PRS. Data represent the mean value (±1 SD) of the PRS for each population. VST is the phenotypic differentiation coefficient (with 95% confidence intervals).

Unadjusted

Adj.-1000G-PCs

V_ST_=0.036 (0.025, 0.047)

V_ST_=0.682 (0.665, 0.699)

Abbreviations: AFR African, AMR Admixed American, EAS East Asian, EUR European, MPI Mariana Pacific Islander, and SAS South Asians.

**Supplemental Figure 7**. Distribution of DIAGRAM 2018 PRS. Data represent the mean value (±1 SD) of the PRS for each population. VST is the phenotypic differentiation coefficient (with 95% confidence intervals).

Adj.-1000G-PCs

Unadjusted

V_ST_=0.002 (-0.001, 0.006)

V_ST_=0.133 (0.114, 0.152)

Abbreviations: AFR African, AMR Admixed American, EAS East Asian, EUR European, MPI Mariana Pacific Islander, and SAS South Asians.

Unadjusted

Adj.-1000G-PCs

Adj.-Study-PCs

V_ST_=0.000 (-0.004, 0.004)

V_ST_=0.034 (0.020, 0.048)

V_ST_=0.051 (0.032, 0.069)

**Supplemental Figure 8.** Distribution of Mars 2022 PRS-Guam subpopulations. Data represent the mean value (±1 SD) of the PRS for each population. VST is the phenotypic differentiation coefficient (with 95% confidence intervals)

V_ST_=0.001 (-0.004, 0.002)

V_ST_=0.004 (-0.002, 0.011)

V_ST_=0.003 (-0.002, 0.009)

Adj.-Study-PCs

Unadjusted

Adj.-1000G-PCs

# **Supplemental Figure 9.** Distribution of Suzuki 2024 PRS-Guam subpopulations. Data represent the mean value (±1 SD) of the PRS for each population. VST is the phenotypic differentiation coefficient (with 95% confidence intervals).

V_ST_=0.000 (-0.004, 0.004)

V_ST_=0.000 (-0.004, 0.003)

V_ST_=0.001 (-0.004, 0.002)

Adj.-Study-PCs

Unadjusted

Adj.-1000G-PCs

# **Supplemental Figure 10.** Distribution of DIAMANTE 2022 PRS-Guam subpopulations. Data represent the mean value (±1 SD) of the PRS for each population. VST is the phenotypic differentiation coefficient (with 95% confidence intervals).

V_ST_=0.000 (-0.004, 0.004)

V_ST_=0.048 (0.030, 0.067)

Unadjusted

Adj.-1000G-PCs

Adj.-Study-PCs

V_ST_=0.038 (0.021, 0.056)

**Supplemental Figure 11.** Distribution of Prive 2022 PRS-Guam subpopulations. Data represent the mean value (±1 SD) of the PRS for each population. V_ST_ is the phenotypic differentiation coefficient (with 95% confidence intervals).

V_ST_=0.022 (0.008, 0.035)

V_ST_=0.000 (-0.004, 0.003)

Adj.-Study-PCs

Unadjusted

Adj.-1000G-PCs

V_ST_=0.029 (0.015, 0.042)

# **Supplemental Figure 12.** Distribution of Khera 2018 PRS-Guam subpopulations. Data represent the mean value (±1 SD) of the PRS for each population. V_ST_ is the phenotypic differentiation coefficient (with 95% confidence intervals).

V_ST_=0.009 (-0.000, 0.018)

V_ST_=0.002 (-0.004, 0.008)

V_ST_=0.000 (-0.004, 0.003)

Adj.-Study-PCs

Adj.-1000G-PCs

Unadjusted

# **Supplemental Figure 13.** Distribution of DIAGRAM 2018 PRS-Guam subpopulations. Data represent the mean value (±1 SD) of the PRS for each population. VST is the phenotypic differentiation coefficient (with 95% confidence intervals).
